# Case of Utero-Vaginal Rupture, during Labour

**Published:** 1841-04-03

**Authors:** J. B. Biddle


*Case of Utero-Vaginal Rupture, during Labour.* By J. B. Biddle, M. D.

S. M., twenty-two years of age, was taken in labour with her second child, Friday, 16th March, at about four o'clock, P. M. She had had a child, about two years previously, born dead, after, as she said, a very severe labour of two days. I was called to her at about half-past eight, P. M. Found the os uteri about the size of a dollar, head presenting. The bowels not having been opened during the day, ordered an injection, which operated. Upon my return at eleven, P. M., the os uteri was well dilated, the membranes ruptured, and the head presenting the occiput to the left acetabulum. The scalp protruded, so as to indicate considerable compression of the head, and I was induced, from the small size of the pelvis and the history of the previous labour, to anticipate a tedious affair. Up to four, A. M., the head made slow but actual progress, from which period the pains, apparently severe, were utterly inefficient in advancing it. The head seemed absolutely jammed in the pelvis, owing, as it was conjectured, to a departure of the chin from the breast. At 12 o'clock, finding that the labour made no progress, I administered (upon consultation with Dr. Wallace,) twenty drops of the wine of ergot, in the hope that more efficient contractions of the uterus might perhaps mould the head of the child to the cavity in which it was impacted, in such a way as to permit its passage. The ergot had no effect. At half-past one, it was determined to atttempt delivery by the forceps, and after unsuccessful efforts to apply the instrument, at half-past two Dr. Meigs was called to the case, which he saw at about three. Dr. Meigs made repeated but unavailing efforts to adjust the forceps upon the head, but it was found impracticable to accomplish the proper apposition of both blades of the instrument. So absolutely did the head fit into the cavity of the pelvis, that it was not possible to introduce the smallest sized catheter into the bladder. At 4 o'clock the pulse being moderately full and strong, eight ounces of blood were taken from the arm, a scruple of powdered ergot was afterwards administered, and the efforts were then unsuccessfully renewed. At six, the pains being strong, forty drops of laudanum were administered, and the patient was left in bed to be seen again at eight o'clock. At that hour it was found that the alternate contractions of the uterus had entirely ceased, her labour pains having left her, as she informed us, shortly after our departure. Contemporaneous with the disappearance of the labour pains was the occurrence of pain in the epigastric region, followed by vomiting. The attempts to apply the forceps were now renewed by Dr. Meigs, and cautiously for a short time continued, but without success in effecting the apposition of the blades. It appearing now finally impracticable to accomplish the delivery with the forceps, and inferring, from auscultation, the death of the child, it was decided at ten o'clock to resort to embryotomy. This was accordingly performed by Dr. Meigs, and the fœtus with very great difficulty extricated, a period of two hours elapsing before it was finally extracted. Even after the entire cerebral mass and the greater portion of the cranium had been removed, the resistance offered to the advancement of the fœtus was most obstinate, proceeding either from the firm contraction of the uterus round the body of the child, or, most probably, as it was surmised, (from palpation over the abdomen and the previous sudden disappearance of the labour pains,) from the escape of the body through a rupture in the uterus which tightly clasped it. The delivery was finally accomplished, literally by main force. Dr. Wallace and myself alternately making traction upon the neck of the child, while Dr. Meigs continued similar efforts with an embryotomy forceps attached to the base of the skull. These efforts at length effected the delivery of the head, that of the shoulders, however, not following without considerable resistance. The size of the child was moderate.—The uterus contracted well and the afterbirth was at once removed. It occurred to Dr. Meigs that his finger on the lower portion of the uterine surface encountered a portion of intestine, confirming the previous conjecture that the uterus had been lacerated. Very trifling external hæmorrhage followed the delivery. The patient was greatly exhausted, and suffered from occasional vomiting. Brandy toddy, volatile julap, and an opiate were administered, and we left the patient at two o'clock somewhat recovered.

Found her next morning free from pain or vomiting; pulse very frequent and feeble; abdomen somewhat distended; had not slept more than half an hour. Thirty drops of laudanum, brandy toddy, beef tea. In the afternoon there was very considerable tympanitis; respiration disturbed; pulse 160, feeble; tongue clean and moist; the beef tea and toddy had not been retained; suppression of urine; no passage from the bowels. Drew off water; administered a scruple of calomel with a grain of opium, followed by half an ounce of castor oil, with ten drops of oil of turpentine. I also introduced a stomach tube per anum, in hopes of procuring a discharge of gas, but without success; then gave an injection of castile soap in tepid water, which brought away a considerable quantity of gas, with a trifling portion of fœcal matter. A good deal of wind was also eructated from the stomach.

Saw her at ten P. M. Symptoms not ameliorated. At twelve, ten grains of calomel were given in half an ounce of castor oil, followed by the black draught, (an infusion of senna, salts, and manna,) which towards morning, produced two tolerably free fœcal evacuations, accompanied with much discharge of wind.

Tuesday, half-past eight, A. M. No better; pulse, 160, very feeble; respiration, 38; tongue tolerably clean; countenance not distressed; abdomen very much distended, but less tympanitic; complains of no pain; no vomiting; lochial discharge; suppression of urine, which was drawn off by a catheter. Ten grains of calomel followed by black draught.

Noon. The medicine had begun to operate. Catheterism.

Half-past three, P. M.—Patient apparently sinking; pulse a mere thread, cannot be counted; respiration very laborious; tongue somewhat coated; great distention of the abdomen; no urine passed. Volatile julap with opium, and brandy toddy.

Eight, P. M.—Slight reaction of the pulse; no passage from the bowels or bladder. A scruple of calomel, and six grs. of Dover's powder every two hours till an operation. Catheterism.

*Eleven, P. M.*—Very restless; flighty; saw her again at three, P. M., when she was evidently moribund; and at six, A. M., she died.

*Autopsy, twelve hours after death.* Present, Drs. Meigs, Wallace, and the reporter. Upon laying open the abdomen, which was enormously distended with gas, the presence of coagulated blood in the abdominal cavity seemed at once to verify the diagnosis of a rupture of the uterus. There was intense peritoneal inflammation, which had advanced to adhesion. Growing from the fundus of the uterus was a small fibro-cartilaginous tumour. The uterus was well contracted. Upon laying it open, a laceration was found at the left posterior portion of the juncture of the uterus with the vagina, through which the body of the child had probably escaped, and being firmly clasped in it, occasioned the difficulty in the delivery which had been encountered. Tissue of the uterus healthy. The antero-posterior diameter of the superior strait found not to exceed three and a quarter inches.

The formidable accident which complicated this labour was of course owing to the obstacle opposed to the delivery by the small size of the pelvis, combined with a probable mal-position of the child's head. The contractions of the uterus, u.able to advance the head, finally forced it through the uterine tissue. The situation of the rupture was the same as in one of two cases reported by Dr. Warrington in this journal. If embryotomy had been resorted to immediately after the head had ceased to advance, the life of the mother would possibly have been saved; but, in a patient who had had a previous natural labour, this operation would have been wholly unjustifiable, during the life of the child, until proper efforts had been made to deliver with the forceps. The efforts to apply them were continued perseveringly, but cautiously, until their failure, and the death of the child, warranted a resort to embryotomy.

Though accidents of this nature have been considered rare, there prevails a strong impression that they are much more frequent complications of labour than the records of obstetrics lead us to suppose. Believing that each isolated case is of value, I have reported the foregoing.

